# Clinical implementation of advanced respiratory monitoring with esophageal pressure and electrical impedance tomography: results from an international survey and focus group discussion

**DOI:** 10.1186/s40635-024-00686-9

**Published:** 2024-10-21

**Authors:** Jantine J. Wisse, Gaetano Scaramuzzo, Mariangela Pellegrini, Leo Heunks, Thomas Piraino, Peter Somhorst, Laurent Brochard, Tommaso Mauri, Erwin Ista, Annemijn H. Jonkman

**Affiliations:** 1 Adult Intensive Care, Erasmus Medical Center, Dr. Molewaterplein 40, 3015GD Rotterdam, The Netherlands; 2 Department of Neonatal and Pediatric Intensive Care, Division of Neonatology, Erasmus Medical Center, Sophia Children’s Hospital, Rotterdam, The Netherlands; 3 Department of Translation Medicine, University of Ferrara, Ferrara, Italy; 4 Department of Emergency, Azienda Ospedaliera Universitaria Sant’ Anna, Ferrara, Italy; 5 Department of Surgical Sciences, Anaesthesiology and Intensive Care Medicine, Uppsala University, Uppsala, Sweden; 6 Department of Intensive Care, Radboud University Medical Center, Nijmegen, The Netherlands; 7 Department of Anesthesia, McMaster University, Hamilton, ON Canada; 8 Keenan Centre for Biomedical Research, Li Ka Shing Knowledge Institute and St. Michael’s Hospital, Unity Health Toronto, Toronto, ON Canada; 9 Interdepartmental Division of Critical Care Medicine, University of Toronto, Toronto, ON Canada; 10 Department of Anesthesia, Critical Care and Emergency, Fondazione IRCCS Ca’ Granda Ospedale Maggiore Policlinico, Milan, Italy; 11 Department of Internal Medicine, Division of Nursing Science, Erasmus Medical Center, Rotterdam, The Netherlands; 12 Department of Neonatal and Pediatric Intensive Care, Division Pediatric Intensive Care, Erasmus Medical Center, Sophia Children’s Hospital, Rotterdam, The Netherlands

**Keywords:** Electrical Impedance Tomography (EIT), Esophageal pressure measurements (Pes), Survey, Panel discussion, Implementation, Barriers and facilitators, Advanced respiratory monitoring

## Abstract

**Background:**

Popularity of electrical impedance tomography (EIT) and esophageal pressure (Pes) monitoring in the ICU is increasing, but there is uncertainty regarding their bedside use within a personalized ventilation strategy. We aimed to gather insights about the current experiences and perceived role of these physiological monitoring techniques, and to identify barriers and facilitators/solutions for EIT and Pes implementation.

**Methods:**

Qualitative study involving (1) a survey targeted at ICU clinicians with interest in advanced respiratory monitoring and (2) an expert focus group discussion. The survey was shared via international networks and personal communication. An in-person discussion session on barriers, facilitators/solutions for EIT implementation was organized with an international panel of EIT experts as part of a multi-day EIT meeting. Pes was not discussed in-person, but we found the focus group results relevant to Pes as well. This was confirmed by the survey results and four additional Pes experts that were consulted.

**Results:**

We received 138 survey responses, and 26 experts participated in the in-person discussion. Survey participants had diverse background [physicians (54%), respiratory therapists (19%), clinical researchers (15%), and nurses (6%)] with mostly > 10 year ICU experience. 84% of Pes users and 74% of EIT users rated themselves as competent to expert users. Techniques are currently primarily used during controlled ventilation for individualization of PEEP (EIT and Pes), and for monitoring lung mechanics and lung stress (Pes). EIT and Pes are considered relevant techniques to guide ventilation management and is helpful for educating clinicians; however, 57% of EIT users and 37% of Pes users agreed that further validation is needed. Lack of equipment/materials, evidence-based guidelines, clinical protocols, and/or the time-consuming nature of the measurements are main reasons hampering Pes and EIT application. Identified facilitators/solutions to improve implementation include international guidelines and collaborations between clinicians/researcher and manufacturers, structured courses for training and use, easy and user-friendly devices and standardized analysis pipelines.

**Conclusions:**

This study revealed insights on the role and implementation of advanced respiratory monitoring with EIT and Pes. The identified barriers, facilitators and strategies can serve as input for further discussions to promote the development of EIT-guided or Pes-guided personalized ventilation strategies.

**Supplementary Information:**

The online version contains supplementary material available at 10.1186/s40635-024-00686-9.

## Background

Advanced respiratory monitoring techniques, such as electrical impedance tomography (EIT) and esophageal pressure (Pes) measurements, allow to monitor the patient’s respiratory response to different mechanical ventilator settings, therapies, or clinical evolution. These techniques could facilitate personalized patient management and a lung- and respiratory-muscle protective ventilation approach [1–3]. Nonetheless, despite decades of physiological studies, routine clinical implementation of both techniques is lacking [3–5].

EIT measures impedance changes in the thorax resulting from small currents applied through a belt with electrodes [5] and is currently the only non-invasive and real-time bedside technique that can visualize regional lung mechanics and the distribution of ventilation [6]. Pes measurement involves the insertion of a dedicated esophageal balloon catheter to obtain a surrogate for pleural pressure and thereby provides insight into the mechanical properties of the respiratory system by separating chest wall and lung mechanics, and quantifies inspiratory effort [7].

Although both techniques provide physiological information, there is an uncertainty regarding which EIT- or Pes-derived parameter to use for which clinical scenario, and how to interpret findings in the complex context of the critically ill throughout the course of ICU admission, both in mechanically ventilated and non-intubated patients. Therefore, evidence and consensus of EIT-guided or Pes-guided ventilation strategies leading to better outcomes are still in an early stage [8–10].

Nevertheless, despite evidence of possible utility, real-life factors, such as need for specific training, equipment, technical limitations or economic issues, may also hinder the clinical implementation of both techniques. To date, no systematic investigation on these issues has been conducted.

Understanding the current landscape of Pes and EIT worldwide can pinpoint gaps between potential use and its actual clinical implementation and may thus be instrumental in guiding future developments. Overcoming implementation barriers will facilitate the conduct and reproducibility of clinical trials and hence, the sustainable implementation of EIT and Pes as accepted valuable tools for individualization of ventilation management. We therefore developed an international survey targeted at ICU clinicians with interest in advanced respiratory monitoring and organized a focus group discussion of expert with the primary aim to describe the current experiences and perceived role of EIT and Pes in clinical practice. Secondary objectives are to identify barriers and facilitators for EIT and Pes implementation.

## Methods

Research ethics approval and pre-registration of the study protocol were not applicable as no patients were involved. The study consists of two independent phases: (1) an online electronic survey directed to ICU clinicians (e.g., physicians, respiratory therapists, nurses, clinical researchers) involved in the management of critically ill patients and (2) focus group discussion including respiratory monitoring experts to explore barriers and facilitators/strategies for implementation of EIT. Participation to the survey and focus group discussion was voluntary; consent was implied through the return of the survey or upon attendance. All responses were analyzed confidentially and anonymously and there was no reward for participation.

### Development of survey for ICU clinicians

Iterative survey development and testing were done by the authors (JW, AJ, MP, GS, PS, and EI). We used the Nonadoption, Abandonment, Scale-up, Spread and Sustainability framework [11] for technologies within a healthcare system to identify the domains to question (which are: knowledge on technology, use cases for which condition/illness, perceived added clinical value, adoption in clinical workflow, and organization). The extent of data collection and questions was discussed, balancing the time for survey completion. Response formats included both binary (yes/no), and ordinal (e.g., Likert scale: ranging from strongly disagree to strongly agree) measurements and open formats (free text). The survey (in English) was built in CastorEDC and reviewed and facilitated by the data capture team of the Erasmus Medical Center. Pilot testing and reviewing was done by survey developers, members of their research team and external experts (totaling *n* = 10) to evaluate survey time, user-friendliness and to finalize questions. The final questionnaire can be found in the online supplemental file 1 and took a maximum of about 15–20 min to complete (depending on whether the participant uses or not Pes and/or EIT).

The survey was open from January 22, 2024 to May 17, 2024 and targeted clinicians and researchers with interest in advanced respiratory monitoring; prior use of Pes and/or EIT was not required. Specific follow-up questions (related to EIT and/or Pes use-cases, competence and experiences, and perceived barriers and facilitators for implementation) appeared according to the respondent’s answer on whether he/she uses EIT and/or Pes in daily practice. The questionnaire with cover letter was shared via e-mail with the Pleural Pressure Working Group (PLUG, www.plugwgroup.org), the EIT-mailing-list (an international mailing-list to promote communication within the community of scientists and clinicians working with EIT), the participants of the focus group discussion, and via personal communication and social media (e.g., LinkedIn). Demographics and initial follow-up questions were analyzed for potential duplicates in anonymous responses (to screen for multiple questionnaires being completed by the same clinician).

### Focus group discussion of EIT experts

We organized a 2.5-h in-person focus group discussion on EIT implementation with an international panel of EIT experts (*n* = 26) as part of a multi-day EIT meeting facilitated by the Lorentz center in Leiden, the Netherlands (April 2024). Participants were invited to the meeting via a personal invitation from the organizational committee, considering their professional background, career level and country to achieve a diverse representation. The focus group discussion was led by a science implementation expert (EI). Barriers and facilitators (determinants) for EIT implementation were identified within the following domains: technological innovation (e.g. evidence, complexity), professional (e.g. attitudes, knowledge/training), organization (e.g. resources, local protocols) and external context (e.g. reimbursement, regulations) [11, 12]. First, experts wrote down their most consequential barriers for EIT use within these domains. The barriers were clustered and served as a starting point for an in-depth discussion in sub-groups per domain led by a moderator. In a second round the groups rotated to discuss a different domain where they focused on facilitators and potential implementation strategies for the barriers identified by the other groups.

Pes application was not part of the in-person discussion; however, we found that the overall identified barriers and facilitators by the focus group were applicable for advanced respiratory monitoring in general. This was evaluated along the survey results and additionally by four experts (TP, TM, LH, and LB) who were consulted to give their opinions on these identified barriers and facilitators within the context of Pes implementation.

## Results

### Participant characteristics and clinical experience

We received a total of 138 unique survey responses within 4 months (response rate unknown but the survey was emailed to > 700 recipients). Survey completeness varied and all completed individual questions related to EIT and/or Pes were included in the analysis. Detailed characteristics of the respondents are presented in Table 1. Most respondents work in mixed adult ICUs (60%), are attending physician (54%), have > 10 years of ICU experience (64%) and are based on Europe (64%). Sixty-three (46%) respondents perform both EIT and Pes measurements in critically ill patients, 42 (30%) respondents only Pes, 13 (9%) respondents only EIT and 20 respondents neither EIT nor Pes (14%). Participants (if being a user of EIT and/or Pes) self-assessed their expertise level from expert to novice. The level of expertise for EIT was expert (28% of EIT users), proficient (22%), competent (24%), advanced beginner (20%) and novice (7%). Pes users qualified their expertise as expert (31%), proficient (30%), competent (23%), advanced beginner (14%) and novice (3%).

**Table 1:** Baseline characteristics of the survey respondents (*n* = 138)

		Number of responses (%)
ICU department^a^	Mixed (adult ICU)	96 (60%)
	Medical (adult ICU)	37 (23%)
	Surgical (adult ICU)	18 (11%)
	Pediatric	9 (6%)
Primary professional background^a^	Attending physician	90 (54%)
	Respiratory therapist	32 (19%)
	Clinical researcher	25 (15%)
	Nurse	10 (6%)
	Physician in training	7 (4%)
	Other clinical role	2 (1%)
	Non-clinical role	2 (1%)
ICU experience (years)	1–3	10 (7%)
	3–5	15 (11%)
	5–10	25 (18%)
	> 10	88 (64%)
Location	Europa	87 (64%)
	North America	27 (20%)
	Asia	10 (7%)
	South America	11 (8%)
	Middle East	1 (1%)
	Australia/New Zealand	1 (1%)
	Africa	0 (0%)
Respondents age (years)	< 30	6 (4%)
	30–39	51 (37%)
	40–49	41 (30%)
	50–59	30 (22%)
	> 60	10 (7%)

^a^Multiple answers could be possible

The focused in-person discussion on EIT implementation included 26 participants from 9 countries (Europe, North America and South America), including clinical doctors, technical physicians, respiratory therapists, and (biomedical) engineers, all with their own interest in or expertise with EIT and ranging from senior (professors) to junior (PhD student) levels. The vast majority of attendants consisted of mid-career clinicians/researchers.

### Clinical value and use cases

Survey respondents who use the techniques were asked about their specific use cases. The primary mentioned reasons to use the techniques are patient monitoring (EIT 34%, Pes 34%), followed by clinical research (EIT 28%, Pes 25%), diagnosing (EIT 22%, Pes 22%) and education (EIT 16%, Pes 19%). Both techniques are currently mostly used for patients during controlled ventilation (EIT 45%, Pes 50%) followed by assisted ventilation (EIT 33%, Pes 40%) and in non-ventilated patients (EIT 23%, Pes 10%).

**Fig. 1:**
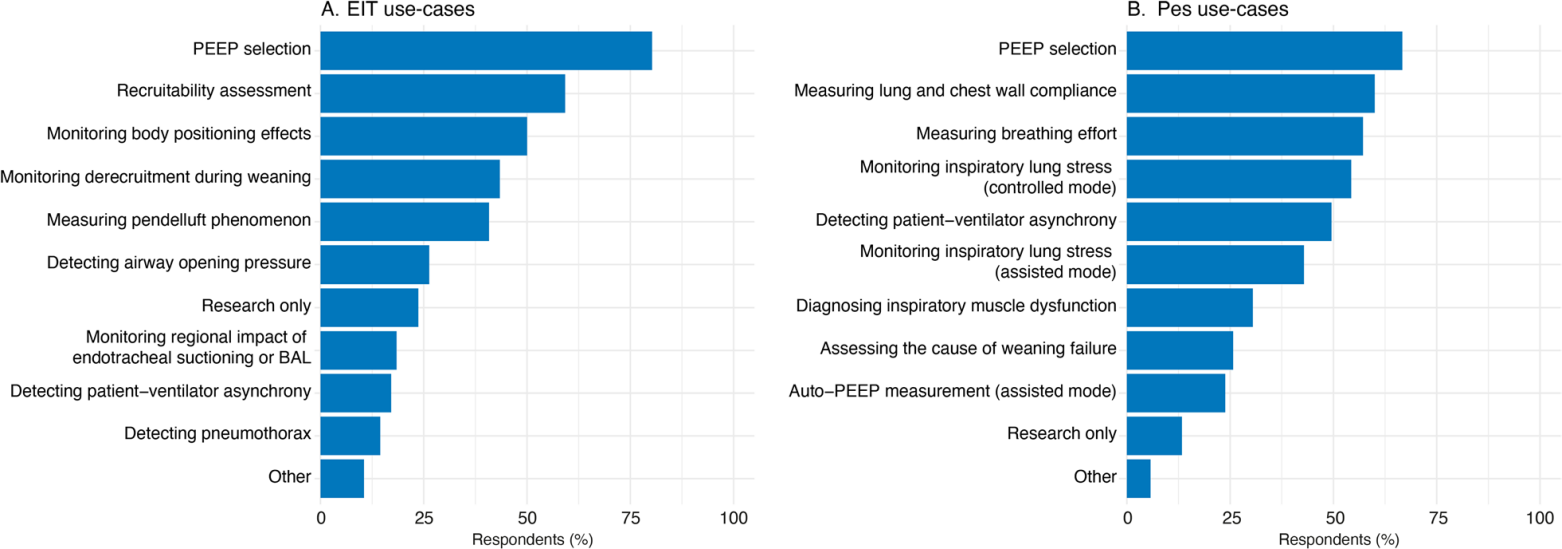
Survey responses to the question: “I use EIT-or-Pes for….”. Survey participants could select the use cases from a predefined list. Multiple use-cases could be selected per measurement

The distribution of use-cases for EIT and Pes is summarized in Fig. 1. The main clinical application for both techniques is selection of the optimal positive end-expiratory pressure (PEEP) level, followed by assessing recruitability with EIT (Fig. 1A) and measuring lung and chest wall compliance with Pes (Fig. 1B). 57% of EIT users and 37% of Pes users found that the technique needs more clinical validation before guiding treatment (i.e., how to set PEEP based on EIT and/or Pes to improve patient outcomes).

Despite being EIT and/or Pes users that inherently recognize added value of the techniques (supplement 2-Fig. 1), most participants did not agree on the statement that it reduces the need for other examinations (mentioned by respectively 63% and 88% of EIT and Pes users).

### Professionals’ training

Respondents mentioned that they learned to perform EIT or Pes measurements in multiple ways. Most participant learned to apply the techniques in clinical care via hands-on training by experts or colleagues (mentioned by 39% of EIT users, 36% of Pes users) or self-training (EIT 31%, Pes 35%), followed by hands-on training from the industry (EIT 20%, Pes 10%) and online courses or masterclasses (EIT 11%, Pes, 18%).

### Organization and team

**Fig. 2:**
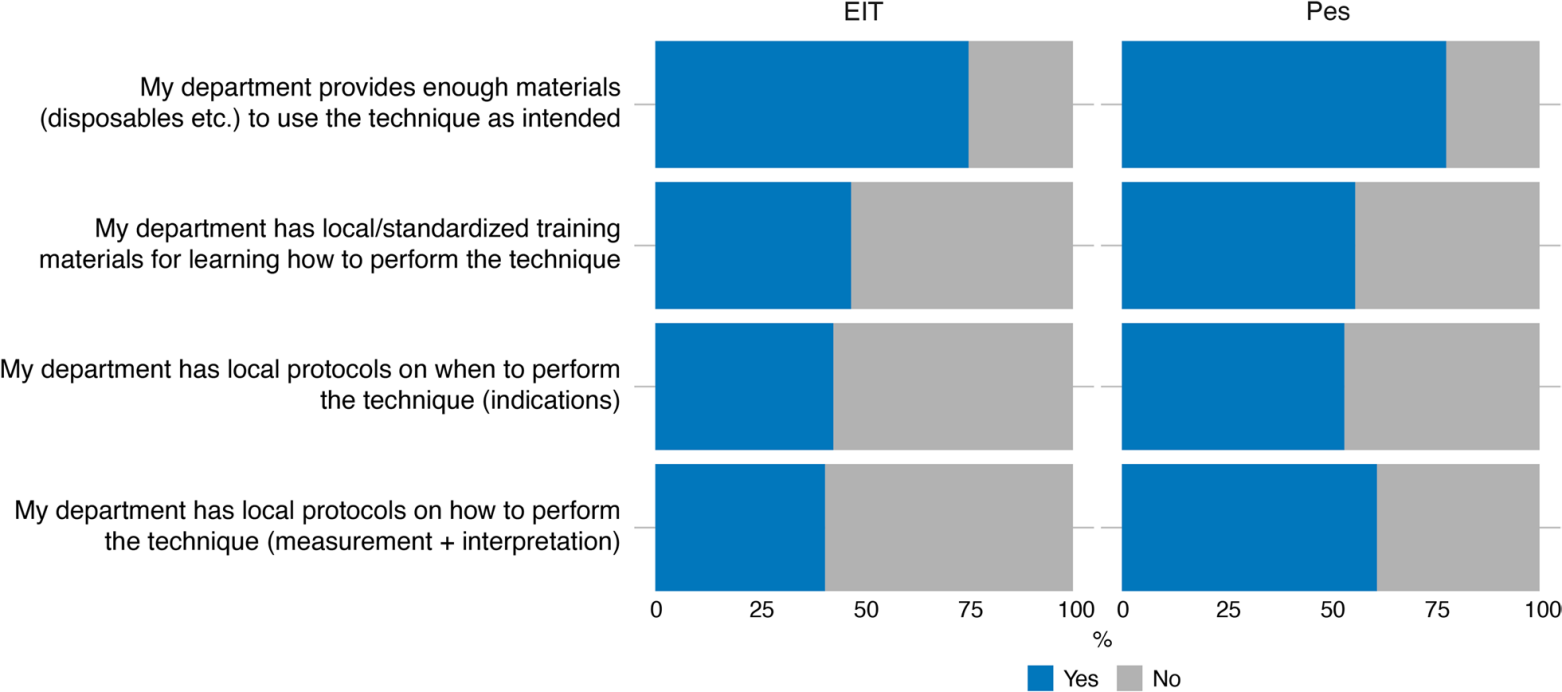
Survey responses related to EIT and Pes use within the organization

Regarding the embedding of EIT and/or Pes monitoring in the team and organization there were two main topics questioned in the survey, including the availability of resources within the department (i.e., materials/equipment), and availability of standardized protocols for use and training. Figure 2 shows a summary of the results. The vast majority of respondents (75% and 78% for EIT and Pes users, respectively) mentioned that their department provides enough materials, e.g., disposable EIT belts (covers), flow sensors or esophageal catheters. Regarding protocols, only between 40 and 50% of respondents mentioned that their department has protocols on training, EIT and/or Pes indications, and how to perform and interpret the measurements.

Most survey participants mentioned that they can discuss their findings with other experts and that they are supported by their colleagues to use the techniques. More than 35% of the respondents indicated that the quality of an EIT or Pes measurement performed by new users is not evaluated, that there are not enough training materials nor people available to perform the measurements, despite having the equipment. 65% of the respondents mentioned that they can incorporate the measurements in their workflow even though a substantial percentage of the respondents (34% of EIT users, 22% of Pes users) disagreed that they have enough time in their day-to-day work to use the techniques.

### Reasons for not using EIT and/or Pes

Participants who responded “No” to the question “Do you perform EIT -or- Pes measurements in critically ill patients?” were subsequently asked to select a reason. The predominant reason for not conducting these measurements was the lack of available equipment (see supplement 2-Fig. 2 for these and other reasons).

Participants who do have EIT and/or Pes techniques available in clinical practice were also asked for the reason why they decide not to use the technique for a patient. The primary reasons were the time-consuming nature of measurements and limited equipment and materials. Approximately 10% of the survey respondents selected either uncertain interpretation or lack of experience as one of the reasons not to perform EIT or Pes.

### Towards improved implementation—barriers and facilitators

**Fig. 3:**
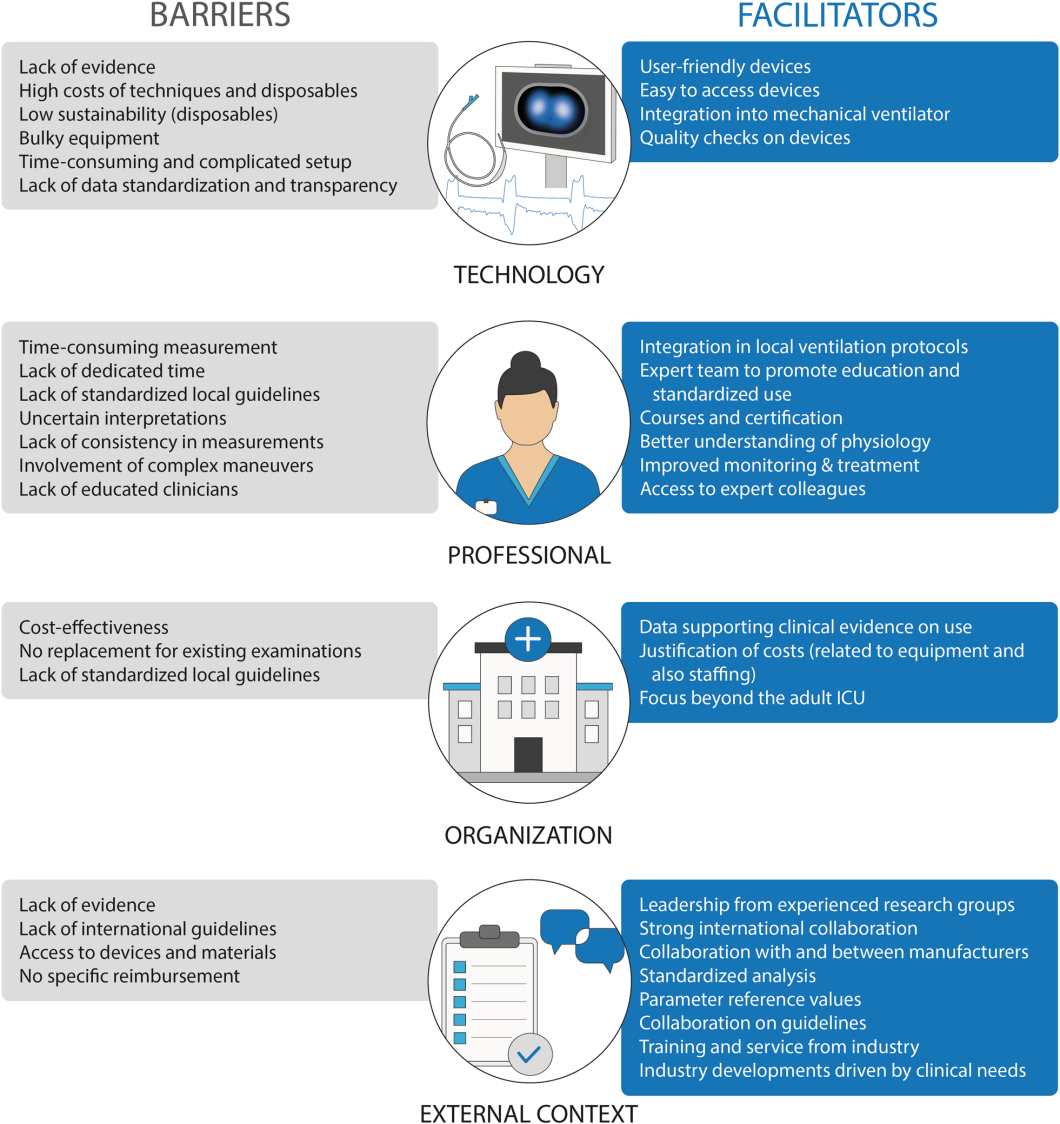
Overview of the barriers and facilitators for the use and implementation of EIT and Pes

Based on the survey and group discussions by clinicians with a particular interest in or expertise with EIT and/or Pes, we identified the most important barriers and facilitators and potential implementation strategies according the four domains (innovation, professionals, organization and external context). These barriers and facilitators are also summarized in Fig. 3.

#### Technological innovation

The lack of evidence of improved patient-centered outcomes was identified as a major barrier for successful clinical implementation of EIT and Pes monitoring. To facilitate wider application of both techniques, experts and survey respondents mentioned that first consensus is needed on how to perform the measurements and how to interpret the results in the clinical context including parameter reference values. Experts argued that it will be challenging to gain evidence with randomized clinical trials on the benefit of EIT and Pes monitoring regarding patient outcomes while there is a lack of standardization of use and interpretation.

Regarding technological innovations, bulky equipment and lack of integration with mechanical ventilators and other devices were mentioned as barriers. More user-friendly devices and materials were identified as a major facilitator. To promote implementation, the following potential strategies were mentioned: (1) synchronization and integration of EIT and Pes within mechanical ventilators, allowing more efficient monitoring. Due to technological advances, Pes measurements can now be integrated in some specific mechanical ventilators; however, for ICUs equipped with other ventilators, there is a lack of dedicated stand-alone Pes monitors beyond research-approved devices. (2) Integration of real-time bedside quality checks into the devices. This was argued to increase the ease of use, confidence of new users, and reliability of (repeated) measurements. (3) The use of both techniques should include simple maneuvers and easy to access and understand numbers (such as occlusion pressure on the ventilator or the measurement of compliance), such that all clinicians can incorporate these techniques in their daily workflow. (4) Allow for sustainable use of disposables, limiting waste and costs.

In addition, the lack of data interoperability between different EIT devices and transparent software was mentioned as a barrier for research and clinical implementation. As a solution, it was argued that standardized open-source analysis pipelines (independent of vendors) could contribute to the generalizability of research and overall implementation.

#### Professionals

A perceived time-consuming nature of the measurements was identified as a potential barrier to implementation. However, it was also argued that the use of EIT and Pes may facilitate longer-term (time-)savings due to an enhanced comprehension of the patient’s respiratory physiology. Experts felt that this is not always recognized by their colleagues or new users, hindering their time-investment needed to gain sufficient expertise. Development of structured courses (potentially with the option to obtain a certificate) and access to expert-peers could improve one’s competence and facilitate adequate bedside use.

In addition, lack of consistency between users in performing measurements and uncertainty about interpretation of findings were identified as a barrier hindering the use of Pes and EIT in the clinical workflow. Again, development of local protocols and access to training materials appeared as a facilitator to enable consistent training and use. It was mentioned that this will help to overcome the identified barrier of not having enough trained users within the department to perform the measurements. However, it was also argued by participants that it may not be necessary to train all ICU clinicians in the use and interpretation of EIT and Pes, considering an already demanding clinical environment. Participants mentioned that a dedicated team could facilitate standardized use and routine implementation; not necessarily being the exclusive group performing measurement, but acting as experts to promote education, provide assistance, and help all clinicians gain experience, thereby ensuring consistency in performing measurements (i.e., comparable to a lung rescue team[13]). If EIT and/or Pes monitoring becomes an integral part of the ventilator management and daily practice, it was recognized that measurements become easier and less time-consuming.

#### Organization

High costs related to the technology and disposables are a barrier for hospitals/departments to implement EIT and/or Pes. In line with the survey results, experts mentioned that EIT and Pes do not necessarily reduce the need for other examinations, making it challenging to justify the costs related to their local implementation, including equipment purchase, disposables and staffing. Participants believed that data supporting the clinical advantages of EIT and Pes is an important facilitator.

When EIT and/or Pes are available within the organization, it was mentioned that local protocols could stimulate adoption into the department’s routine clinical workflow despite the lack of international evidence-based guidelines. These local protocols should outline a step-by-step measurement procedure adapted to the local measurement setup/materials that are available, but also outlining the selection of the appropriate patient population (i.e., when to initiate advanced monitoring) and how to interpret the data.

Finally, it was mentioned that EIT could be of interest to other departments (e.g., pulmonary medicine, emergency medicine, and operating theatre) beyond its current use in the intensive care setting (adult, pediatric and neonatal). Acknowledging the potential of technological innovations for multiple departments could then stimulate the wider adoption of the technique within an organization and substantiate funding decisions.

#### External context

It was perceived that the development of reimbursement schemes for the use of advanced respiratory monitoring technique is hampered by the absence of evidence. This could hamper hospital’s investments in devices and justification of costs. It was also mentioned that, as a consequence, current application of EIT and Pes is mostly limited to top-level ICUs in well-developed countries. Better overall availability of devices and disposables should be pursued.

Experts mentioned that the focus should shift to developing strategies for personalizing mechanical ventilation (i.e., standardized approach for assessing recruitability and optimizing lung mechanics for PEEP setting using EIT and/or Pes). In the absence of evidence-based international guidelines, participants recommended starting with developing local protocols first (see Organization section).

Furthermore, it was mentioned that greater knowledge within the community, including patients and their representatives, about the existence of EIT and Pes and the potential to reduce the need for more invasive diagnostics may promote implementation.

A strong agreement was shown on the need for technological developments by the industry to be driven by clinical needs. Close collaboration between manufacturers and clinicians was therefore considered essential.

## Discussion

Insights from our international survey and focused expert discussions among participants with a particular interest in or expertise with advanced respiratory monitoring on the perceived role, experiences and implementation of EIT and Pes in the ICU can be summarized as follows: (1) EIT and/or Pes users currently mostly apply the techniques during controlled ventilation for individualization of the PEEP level (EIT and Pes) and monitoring of lung mechanics and inspiratory lung stress (Pes) and (2) We identified todays barriers and facilitators for successful implementation of advanced respiratory monitoring techniques. Major barriers are: a lack of equipment and protocols and/or the time-consuming nature of the measurements. In addition, many clinicians find that more clinical validation and standardized evidence-based guidelines are needed. The development of international guidelines and collaborations among clinicians, researchers and manufacturers were identified as important facilitators. Other key facilitators included structured courses for standardized training and use, easy-to-use devices, and standardized analysis pipelines.

Although EIT and Pes have a high perceived potential for optimizing patient care, routine clinical implementation remains a challenge [7, 14]. Assessing barriers and facilitators for the use of advanced respiratory monitoring is a first step towards developing strategies for enhancing local implementation of EIT and Pes and developing international or standardized guidelines [15]. Respondents of the survey agreed that EIT and Pes contribute to a better understanding of the individual patient’s respiratory physiology and that this helps guiding clinical decisions. However, whether EIT and Pes monitoring improves patient treatment and reduces the need for other examinations was less distinctly indicated. Clear protocols on EIT- and Pes-guided ventilation strategies were identified as facilitators for their clinical use. Indeed, protocols on particular aspects of care have been associated with improved outcomes for critically ill patients [16]. On the other hand, when EIT and Pes tools are primarily used to better understand respiratory physiology, the need for such protocols can be questioned. For instance, the current perceived role for EIT and Pes monitoring is mostly for setting PEEP. Whereas EIT could help to identify if a patients is recruitable [17], the classification of the patient as one of the sub-phenotypes (recruitable yes/not), not the technology per se, guides subsequent treatment and ventilation management. Nevertheless, protocols on the use, patient selection and interpretation of EIT and Pes are needed for standardized and routine implementation—which also promotes generalizability of research. An illustrative example is the EIT-based lung overdistention-collapse approach for setting PEEP during a decremental PEEP trial [18]. While this measurement is rather easy to perform at the bedside and provides direct results (trend of %collapse and %overdistension, and their crossing point), there is no consensus on how to perform such PEEP trials in a structured way (i.e., the range of applied PEEP steps mathematically influences the results) and how to select the PEEP based on this method (e.g., at crossing point, minimal overdistension, etc.) [19, 20].

International training facilities were mentioned as a facilitator for EIT and Pes application. Different statement papers describing the meaning, usefulness and perspective of the EIT and/or Pes in clinical settings are yet available [3, 7, 21, 22] initiated or facilitated by international working groups (PLUG/ESICM and/or ERS), as well as training materials in the form of videos (e.g., plugwgroup.org). However, whereas for ultrasound application many international training facilities are available, this is less well-developed for EIT and Pes. Strong leadership from research groups could help to set up international courses and to integrate advanced respiratory monitoring in international programs such as EDIC (European Diploma in Intensive Care) and thereby increase knowledge within the ICU community. Including simulation training in the development of courses might help to further improve skills in advanced respiratory management compared to traditional courses [23]. Costs, availability of courses and the potential of certification should be carefully considered to not hinder the clinical use of Pes and EIT—which should ideally be accessible to all ICU clinicians.

### Strengths and limitations

This study has some strengths and limitations. To the best of our knowledge this was the first survey providing implementation barriers and facilitators for advanced respiratory monitoring. A previous survey was conducted among 32 clinicians on the perceived usefulness of measures derived from EIT examinations in neonatology and pediatrics. This survey found that EIT measures characterizing the ventilation and aeration distribution and heterogeneity were deemed particularly useful [24].

Our survey was conducted among a large international group, representing different backgrounds and cultures, and with various self-reported levels of expertise. Most respondent were from Europe, with Asian respondents being underrepresented despite the perceived increasing clinical use and device development in Asian countries. Response rate could not be assessed. Since participation was promoted via the PLUG and EIT networks, a bias (i.e., positive attitude towards both Pes and EIT) is likely present and the results reflect the opinion of clinicians with particular interest in these techniques. A variety in years of ICU experience and expertise with EIT and/or Pes among participants was represented in both the survey responses and focus group discussion. Nevertheless, the survey was also completed by respondents that do not use EIT and/or Pes in clinical practice and their reasons not to use the techniques were collected. However, it would have been interesting to also more extensively collect their perceived barriers for implementation of advanced respiratory monitoring.

Pes application was not part of the focus group discussion. The reported barriers and facilitators regarding Pes implementation resulted from the survey and were separately evaluated by experts (TP, TM, LH, and LB). We did not use a standardized methodology such as the Delphi method [25] to generate consensus on the clinical use of EIT and Pes, but performed a rather qualitative assessment. Nevertheless, we used a structured framework (NASSS [11]) and the EIT focus group discussion was led by a science implementation expert (EI) who was also involved in survey development.

## Conclusion

Our international survey and panel discussion reveals insights on the role and implementation of advanced respiratory monitoring with EIT and Pes. Currently, both techniques are primarily used during controlled ventilation to individualize PEEP levels, followed by assessing lung mechanics and inspiratory stress with Pes. Barriers for implementation that were identified included the lack of equipment and protocols and the time-consuming nature of measurements, and as well as the need for more clinical validation and evidence-based guidelines. Various facilitators/solutions for improved implementation were identified, including international guidelines and collaborations between clinicians and researcher but also manufacturers, structured courses for standardized training and use, easy and user-friendly devices and standardized analysis pipelines. These solutions provide future perspectives and will likely promote the development of EIT-guided or Pes-guided personalized ventilation strategies, and eventually improve patient outcomes.

## Supplementary Information

Supplementary material 1.

Supplementary material 2.

## Data Availability

The datasets used and/or analysed during the current study are available from the corresponding author on reasonable request.

## Abbreviations

EDIC: European Diploma in Intensive Care
EIT: Electrical impedance tomography
ERS: European Respiratory Society
ESICM: European Society of Intensive Care Medicine
ICU: Intensive Care Unit
NASSS: Nonadoption, abandonment, scale-up, spread, and sustainability
Pes: Esophageal pressure
PEEP: Positive end expiratory pressure
PLUG: Pleural Pressure working group
