# Supplementary material for: Clinical implementation of advanced respiratory monitoring with esophageal pressure and electrical impedance tomography: results from an international survey and focus group discussion
Source: Intensive Care Med Exp. 2024 Oct 21;12:93. doi: 10.1186/s40635-024-00686-9 (PMC11493933; doi:10.1186/s40635-024-00686-9)
Supplement: Supplementary file 1 — Supplementary material 1. [file 40635_2024_686_MOESM1_ESM.pdf]

Surveys of Pes_EIT_survey - version 54.41

Printed on 16-07-2024 09:04:42 by Jantine Wisse - Smit

Survey 'Use and implementation of advanced respiratory monitoring (Pes and EIT) in the ICU'

Use and implementation of advanced respiratory monitoring (Pes and EIT) in the ICU - Introduction

Good day!

Thank you for your willingness in participating in this survey!

Practical notes:

- The survey will take about 15 minutes to complete, or less if you do not use both Pes and EIT. Questions on next pages appear according to your answers on whether you use Pes and/or EIT (first page, questions 6 and 7).
- This survey is in English. Changing the language settings will only change the 'Previous'/'Next' buttons.

Your participation to the survey will make a difference and will help us individualizing mechanical ventilation and improving patients' outcomes.

Why did we create this survey?

As you may know, advanced respiratory monitoring through **electrical impedance tomography (EIT)** and **oesophageal pressure (Pes)** measurements is increasingly employed in the critically ill as it provides additional information of the individual patient's respiratory physiology. Hence, clinicians can monitor the patient's respiratory response to different mechanical ventilator settings, therapies, or clinical evolution, **allowing a more personalized mechanical ventilation approach.**

Both techniques provide a **wealth of valuable information**, but there is a **remaining uncertainty regarding the clinical use cases and how to interpret findings** in the context of the critically ill and throughout the course of mechanical ventilation. As a consequence, evidence of EIT-guided or Pes-guided ventilation strategies leading to better outcomes is still in its infancy.

Objectives

With this survey, **we aim to gather insight about the current experiences and perceived role of EIT and/or Pes in clinical practice.** Secondary objectives are to describe the perceived clinical and practical challenges and factors that facilitate or hinder the implementation of these advanced respiratory monitoring techniques.

Outcomes

We will use the outcomes of this survey as input for **further discussions on the future role of Pes and EIT** and how to move towards developing and implementing **mechanical ventilation strategies guided by advanced respiratory monitoring.**

Thank you very much.

Best regards,

Annemijn Jonkman, Mariangela Pellegrini, Gaetano Scaramuzzo, Jantine Wisse-Smit, Peter Somhorst and Erwin Ista

Use and implementation of advanced respiratory monitoring (Pes and EIT) in the ICU - Background and experience with EIT and Pes

Number	Question	Answers
1.1	In which type of ICU do you work?<br>multiple options possible	☐ medical (adult ICU)<br>☐ surgical (adult ICU)<br>☐ mixed (adult ICU)<br>☐ pediatric
1.2	What is your professional background?<br>multiple options possible (e.g., physician in training and clinical researcher)	☐ attending physician<br>☐ physician in training<br>☐ respiratory therapist<br>☐ nurse<br>☐ other clinical role<br>☐ clinical researcher<br>☐ non-clinical role
1.3	What is your age group?	○ <30 years old<br>○ 30-39 years old<br>○ 40-49 years old<br>○ 50-59 years old<br>○ >60 years old

1.4	How many years of experience in the ICU do you have?	◯ 1-3 years<br>◯ 3-5 years<br>◯ 5-10 years<br>◯ >10 years
1.5	Where do you work?	◯ Africa<br>◯ Asia<br>◯ Australia/NZ<br>◯ Europe<br>◯ Middle East<br>◯ North-America<br>◯ South-America
1.6	Do you perform EIT in critically ill patients?	◯ yes<br>◯ no
1.6.1	***If 'Do you perform EIT in critically ill patients?' is equal to 'yes' answer this question:***<br>What level of expertise do you have with EIT? Range from novice ("I have limited knowledge about EIT and I have performed very few EIT examinations”) to expert (“I have extensive knowledge about EIT and I have performed a large amount of EIT examinations”)<br>Range from novice ("I have limited knowledge about EIT and I have performed very few EIT examinations”) to expert (“I have extensive knowledge about EIT and I have performed a large amount of EIT examinations”)	◯ novice ◯ advanced beginner ◯ competent ◯ proficient ◯ expert
1.6.2	***If 'Do you perform EIT in critically ill patients?' is equal to 'yes' answer this question:***<br>How did you learn to perform EIT?	☐ (online) courses or masterclasses<br>☐ hands-on training from industry<br>☐ hands-on training from other experts or colleagues<br>☐ self-training

1.6.3	***If 'Do you perform EIT in critically ill patients?' is equal to 'yes' answer this question:***<br>I perform EIT in<br>multiple options possible	☐ non-ventilated patients<br>☐ patient on controlled ventilation<br>☐ patients on assisted ventilation/during weaning
1.6.4	***If 'Do you perform EIT in critically ill patients?' is equal to 'yes' answer this question:***<br>How often do you use EIT in clinical practice?	○ very often: multiple times a week<br>○ often: once a week<br>○ sometimes: multiple times a month<br>○ rarely: once a month<br>○ never for clinical reasons / only for research
1.6.5	***If 'Do you perform EIT in critically ill patients?' is equal to 'yes' answer this question:***<br>How do you use EIT in clinical practice?<br>multiple options possible	☐ for diagnosing<br>☐ for monitoring<br>☐ for education<br>☐ for clinical research
1.6.6	***If 'Do you perform EIT in critically ill patients?' is equal to 'no' answer this question:***<br>Why do you not use EIT in your clinical practice?<br>multiple options possible	☐ we do not have the equipment available<br>☐ we have too few equipment/materials available<br>☐ we have the equipment but I am not trained enough for using it<br>☐ I am not sure how to interpret the values<br>☐ budget (too expensive to perform)<br>☐ other
1.6.6.1	***If 'Why do you not use EIT in your clinical practice?' is equal to 'other' answer this question:***<br>Please specify 'other'	
1.7	Do you perform Pes measurements in critically ill patients?	○ yes<br>○ no

1.7.1	***If 'Do you perform Pes measurements in critically ill patients?' is equal to 'yes' answer this question:***<br>What level of expertise do you have with oesophageal pressure (Pes) measurements?<br>Range from novice ("I have limited knowledge about Pes and I have performed very few Pes examinations”) to expert (“I have extensive knowledge about Pes and I have performed a large amount of Pes examinations”)<br>Range from novice ("I have limited knowledge about Pes and I have performed very few Pes examinations”) to expert (“I have extensive knowledge about Pes and I have performed a large amount of Pes examinations”)	○ novice ○ advanced beginner ○ competent ○ proficient ○ expert
1.7.2	***If 'Do you perform Pes measurements in critically ill patients?' is equal to 'yes' answer this question:***<br>How did you learn to perform Pes measurements?	☐ (online) courses or masterclasses<br>☐ hands-on training from industry<br>☐ hands-on training from other experts or colleagues<br>☐ self-training
1.7.3	***If 'Do you perform Pes measurements in critically ill patients?' is equal to 'yes' answer this question:***<br>I perform Pes measurements in<br>multiple options possible	☐ non-ventilated patients<br>☐ patient on controlled ventilation<br>☐ patients on assisted ventilation/during weaning
1.7.4	***If 'Do you perform Pes measurements in critically ill patients?' is equal to 'yes' answer this question:***<br>How often do you use Pes in clinical practice?	○ very often: multiple times a week<br>○ often: once a week<br>○ sometimes: multiple times a month<br>○ rarely: once a month<br>○ never for clinical reasons / only for research
1.7.5	***If 'Do you perform Pes measurements in critically ill patients?' is equal to 'yes' answer this question:***<br>How do you use Pes in clinical practice?<br>multiple options possible	☐ for diagnosing<br>☐ for monitoring<br>☐ for education<br>☐ for clinical research

1.7.6	***If 'Do you perform Pes measurements in critically ill patients?' is equal to 'no' answer this question:***<br>Why do you no not use Pes in your clinical practice?<br>multiple options possible	☐ we do not have the equipment available<br>☐ we have too few equipment/materials available<br>☐ we have the equipment but I am not trained enough for using it<br>☐ I am not sure how to interpret the values<br>☐ budget (too expensive to perform)<br>☐ other
1.7.6.1	***If 'Why do you no not use Pes in your clinical practice?' is equal to 'other' answer this question:***<br>Please specify 'other'	

Use and implementation of advanced respiratory monitoring (Pes and EIT) in the ICU - EIT - perceived clinical role and use cases

Number	Question	Answers
	**The following statements are about your perceived role of EIT. For all statements, fill in what is applicable to EIT in your opinion.**	
2.1	***If 'Do you perform EIT in critically ill patients?' is equal to 'yes' answer this question:***<br>I find that EIT helps me diagnose several conditions	○ totally agree ○ agree ○ neither agree nor disagree ○ disagree ○ totally disagree
2.2	***If 'Do you perform EIT in critically ill patients?' is equal to 'yes' answer this question:***<br>I find that EIT helps me to monitor my patients	○ totally agree ○ agree ○ neither agree nor disagree ○ disagree ○ totally disagree
2.3	***If 'Do you perform EIT in critically ill patients?' is equal to 'yes' answer this question:***<br>I find that EIT helps me in clinical decision making	○ totally agree ○ agree ○ neither agree nor disagree ○ disagree ○ totally disagree

2.4	***If 'Do you perform EIT in critically ill patients?' is equal to 'yes' answer this question:***<br>I find that EIT helps me for education of colleagues	◯ totally agree ◯ agree ◯ neither agree nor disagree ◯ disagree ◯ totally disagree
2.5	***If 'Do you perform EIT in critically ill patients?' is equal to 'yes' answer this question:***<br>I find that EIT helps me to better understand respiratory physiology	◯ totally agree ◯ agree ◯ neither agree nor disagree ◯ disagree ◯ totally disagree
2.6	***If 'Do you perform EIT in critically ill patients?' is equal to 'yes' answer this question:***<br>I find that EIT leads to changes in ventilator management	◯ totally agree ◯ agree ◯ neither agree nor disagree ◯ disagree ◯ totally disagree
2.7	***If 'Do you perform EIT in critically ill patients?' is equal to 'yes' answer this question:***<br>I find that EIT improves my patients' treatment	◯ totally agree ◯ agree ◯ neither agree nor disagree ◯ disagree ◯ totally disagree
2.8	***If 'Do you perform EIT in critically ill patients?' is equal to 'yes' answer this question:***<br>I find that EIT reduces the need for other examinations (e.g., X-thorax, ultrasound)	◯ totally agree ◯ agree ◯ neither agree nor disagree ◯ disagree ◯ totally disagree
	**The following statements are about the clinical use cases of EIT. For all statements, fill in what is applicable to EIT in your opinion.**	
2.9	***If 'Do you perform EIT in critically ill patients?' is equal to 'yes' answer this question:***<br>I think that EIT could be helpful for detecting pneumothorax	◯ totally agree ◯ agree ◯ neither agree nor disagree ◯ disagree ◯ totally disagree
2.10	***If 'Do you perform EIT in critically ill patients?' is equal to 'yes' answer this question:***<br>I think that EIT could be helpful for detecting displacement of the endotracheal tube	◯ totally agree ◯ agree ◯ neither agree nor disagree ◯ disagree ◯ totally disagree
2.11	***If 'Do you perform EIT in critically ill patients?' is equal to 'yes' answer this question:***<br>I think that EIT could be helpful for monitoring the regional impact of endotracheal suctioning or BAL	◯ totally agree ◯ agree ◯ neither agree nor disagree ◯ disagree ◯ totally disagree
2.12	***If 'Do you perform EIT in critically ill patients?' is equal to 'yes' answer this question:***<br>I think that EIT could be helpful for PEEP selection	◯ totally agree ◯ agree ◯ neither agree nor disagree ◯ disagree ◯ totally disagree

2.13	***If 'Do you perform EIT in critically ill patients?' is equal to 'yes' answer this question:*** I think that EIT could be helpful for monitoring derecruitment during weaning	◯ totally agree ◯ agree ◯ neither agree nor disagree ◯ disagree ◯ totally disagree
2.14	***If 'Do you perform EIT in critically ill patients?' is equal to 'yes' answer this question:*** I think that EIT could be helpful for detecting the pendelluft phenomenon	◯ totally agree ◯ agree ◯ neither agree nor disagree ◯ disagree ◯ totally disagree
2.15	***If 'Do you perform EIT in critically ill patients?' is equal to 'yes' answer this question:*** I think that EIT could be helpful for detecting patient-ventilator asynchrony	◯ totally agree ◯ agree ◯ neither agree nor disagree ◯ disagree ◯ totally disagree
2.16	***If 'Do you perform EIT in critically ill patients?' is equal to 'yes' answer this question:*** I think that EIT could be helpful for detecting airway opening pressure	◯ totally agree ◯ agree ◯ neither agree nor disagree ◯ disagree ◯ totally disagree
2.17	***If 'Do you perform EIT in critically ill patients?' is equal to 'yes' answer this question:*** I think that EIT could be helpful for assessing recruitability	◯ totally agree ◯ agree ◯ neither agree nor disagree ◯ disagree ◯ totally disagree
2.18	***If 'Do you perform EIT in critically ill patients?' is equal to 'yes' answer this question:*** I think that EIT could be helpful for monitoring the effect of lateral or prone positioning	◯ totally agree ◯ agree ◯ neither agree nor disagree ◯ disagree ◯ totally disagree

2.19	***If 'Do you perform EIT in critically ill patients?' is equal to 'yes' answer this question:***<br>I use EIT for<br>multiple options possible	☐ detecting pneumothorax<br>☐ detecting displacement of the endotracheal tube<br>☐ monitoring the regional impact of endotracheal suctioning or BAL<br>☐ PEEP selection<br>☐ monitoring derecruitment during weaning<br>☐ detecting the pendelluft phenomenon<br>☐ detecting patient-ventilator asynchrony<br>☐ detecting airway opening pressure<br>☐ assessing recruitability<br>☐ monitoring the effect of lateral or prone positioning<br>☐ research only<br>☐ other
2.19.1	***If 'I use EIT for' is equal to 'other' answer this question:***<br>Please specify other	
	**As you are not using EIT this form is not applicable. Please click 'Next'.**	

Use and implementation of advanced respiratory monitoring (Pes and EIT) in the ICU - EIT - competence, experiences and team/organization

Number	Question	Answers
	**The following statements are about your competence and experience with EIT. For all statements, fill in what is applicable to EIT in your opinion.**	
3.1	***If 'Do you perform EIT in critically ill patients?' is equal to 'yes' answer this question:***<br>It is clear to me how the EIT image is being generated	○ totally agree ○ agree ○ neither agree nor disagree ○ disagree ○ totally disagree

3.2	***If 'Do you perform EIT in critically ill patients?' is equal to 'yes' answer this question:***<br>It is clear to me how I should practically perform an EIT recording in my patient	◯ totally agree ◯ agree ◯ neither agree nor disagree ◯ disagree ◯ totally disagree
3.3	***If 'Do you perform EIT in critically ill patients?' is equal to 'yes' answer this question:***<br>EIT examinations are too complex for me to perform	◯ totally agree ◯ agree ◯ neither agree nor disagree ◯ disagree ◯ totally disagree
3.4	***If 'Do you perform EIT in critically ill patients?' is equal to 'yes' answer this question:***<br>EIT examinations are too time-consuming for me te perform	◯ totally agree ◯ agree ◯ neither agree nor disagree ◯ disagree ◯ totally disagree
3.5	***If 'Do you perform EIT in critically ill patients?' is equal to 'yes' answer this question:***<br>I know how EIT parameters are computed	◯ totally agree ◯ agree ◯ neither agree nor disagree ◯ disagree ◯ totally disagree
3.6	***If 'Do you perform EIT in critically ill patients?' is equal to 'yes' answer this question:***<br>EIT examination are too complex for me to interpret	◯ totally agree ◯ agree ◯ neither agree nor disagree ◯ disagree ◯ totally disagree
3.7	***If 'Do you perform EIT in critically ill patients?' is equal to 'yes' answer this question:***<br>I can incorporate EIT easily in my current workflow	◯ totally agree ◯ agree ◯ neither agree nor disagree ◯ disagree ◯ totally disagree
3.8	***If 'Do you perform EIT in critically ill patients?' is equal to 'yes' answer this question:***<br>I think EIT is relevant for my patients	◯ totally agree ◯ agree ◯ neither agree nor disagree ◯ disagree ◯ totally disagree
3.9	***If 'Do you perform EIT in critically ill patients?' is equal to 'yes' answer this question:***<br>I think the amount of information from EIT can be overwhelming	◯ totally agree ◯ agree ◯ neither agree nor disagree ◯ disagree ◯ totally disagree
3.10	***If 'Do you perform EIT in critically ill patients?' is equal to 'yes' answer this question:***<br>I think that EIT needs more validation before guiding treatment	◯ totally agree ◯ agree ◯ neither agree nor disagree ◯ disagree ◯ totally disagree

3.11	***If 'Do you perform EIT in critically ill patients?' is equal to 'yes' answer this question:*** What are your biggest considerations if you decide not to perform an EIT measurement? multiple options possible	☐ it takes too much time to perform<br>☐ we have too few equipment/materials available<br>☐ I have not enough experience in doing the measurements<br>☐ I am not sure how to interpret the values<br>☐ budget (too expensive to perform (disposables etc.))<br>☐ other<br>☐ not applicable
3.11.1	***If 'What are your biggest considerations if you decide not to perform an EIT measurement?' is equal to 'other' answer this question:*** Please specify 'other'	
	**The following statements are about your team and organization. For all statements, fill in what is applicable to EIT in your opinion.**	
3.12	***If 'Do you perform EIT in critically ill patients?' is equal to 'yes' answer this question:*** My colleagues expect and support me to use EIT	○ totally agree ○ agree ○ neither agree nor disagree ○ disagree ○ totally disagree
3.13	***If 'Do you perform EIT in critically ill patients?' is equal to 'yes' answer this question:*** There are enough people at our department that can perform EIT as intended	○ totally agree ○ agree ○ neither agree nor disagree ○ disagree ○ totally disagree
3.14	***If 'Do you perform EIT in critically ill patients?' is equal to 'yes' answer this question:*** There are enough training materials for new EIT users in our department	○ totally agree ○ agree ○ neither agree nor disagree ○ disagree ○ totally disagree
3.15	***If 'Do you perform EIT in critically ill patients?' is equal to 'yes' answer this question:*** Our team evaluates the quality of EIT recordings made by new users	○ totally agree ○ agree ○ neither agree nor disagree ○ disagree ○ totally disagree
3.16	***If 'Do you perform EIT in critically ill patients?' is equal to 'yes' answer this question:*** I can discuss my EIT findings with other experts when needed	○ totally agree ○ agree ○ neither agree nor disagree ○ disagree ○ totally disagree

3.17	***If 'Do you perform EIT in critically ill patients?' is equal to 'yes' answer this question:*** There is enough time to use EIT in my day-to-day work	◯ totally agree ◯ agree ◯ neither agree nor disagree ◯ disagree ◯ totally disagree
3.18	***If 'Do you perform EIT in critically ill patients?' is equal to 'yes' answer this question:*** Our department provides me with enough materials (disposables etc.) to use EIT as intended	◯ yes<br>◯ no
3.19	***If 'Do you perform EIT in critically ill patients?' is equal to 'yes' answer this question:*** Our department has local or standardized training materials available for learning how to perform EIT (how to apply the belt, position the patient, start the measurement)	◯ yes<br>◯ no
3.20	***If 'Do you perform EIT in critically ill patients?' is equal to 'yes' answer this question:*** Our department has local or standardized protocols on when to perform EIT (indications)	◯ yes<br>◯ no
3.21	***If 'Do you perform EIT in critically ill patients?' is equal to 'yes' answer this question:*** Our department has local or standardized protocols on how to perform EIT (how to do the measurement and interpret results)	◯ yes<br>◯ no
	**The following open questions are about clinical impementation of EIT.**	
3.22	***If 'Do you perform EIT in critically ill patients?' is equal to 'yes' answer this question:*** In your experience, which factors will facilitate the use of EIT in clinical practice?	
3.23	***If 'Do you perform EIT in critically ill patients?' is equal to 'yes' answer this question:*** In your experience, what are the biggest concerns that hinder the use of EIT in clinical practice?	

3.24	***If 'Do you perform EIT in critically ill patients?' is equal to 'yes' answer this question:***<br>(Optional): What do you think should be the next steps towards an EIT-guided ventilation strategy?	
	**As you are not using EIT this form is not applicable. Please click 'Next'.**	

Use and implementation of advanced respiratory monitoring (Pes and EIT) in the ICU - Esophageal pressure measurements - perceived clinical role and use cases

Number	Question	Answers
	**The following statements are about your perceived role of Pes. For all statements, fill in what is applicable to Pes in your opinion.**	
4.1	***If 'Do you perform Pes measurements in critically ill patients?' is equal to 'yes' answer this question:***<br>I find that Pes helps me to monitor my patients	○ totally agree ○ agree ○ neither agree nor disagree ○ disagree ○ totally disagree
4.2	***If 'Do you perform Pes measurements in critically ill patients?' is equal to 'yes' answer this question:***<br>I find that Pes helps me in clinical decision making	○ totally agree ○ agree ○ neither agree nor disagree ○ disagree ○ totally disagree
4.3	***If 'Do you perform Pes measurements in critically ill patients?' is equal to 'yes' answer this question:***<br>I find that Pes helps me for education of colleagues	○ totally agree ○ agree ○ neither agree nor disagree ○ disagree ○ totally disagree
4.4	***If 'Do you perform Pes measurements in critically ill patients?' is equal to 'yes' answer this question:***<br>I find that Pes helps me to better understand respiratory physiology	○ totally agree ○ agree ○ neither agree nor disagree ○ disagree ○ totally disagree
4.5	***If 'Do you perform Pes measurements in critically ill patients?' is equal to 'yes' answer this question:***<br>I find that Pes leads to changes in ventilator management	○ totally agree ○ agree ○ neither agree nor disagree ○ disagree ○ totally disagree

4.6	***If 'Do you perform Pes measurements in critically ill patients?' is equal to 'yes' answer this question:***<br>I find that Pes improves my patients’ treatment	○ totally agree ○ agree ○ neither agree nor disagree ○ disagree ○ totally disagree
4.7	***If 'Do you perform Pes measurements in critically ill patients?' is equal to 'yes' answer this question:***<br>I find that Pes reduces the need for other examinations (e.g., X-thorax, ultrasound)	○ totally agree ○ agree ○ neither agree nor disagree ○ disagree ○ totally disagree
	**The following statements are about the clinical use cases of PES. For all statements, fill in what is applicable to PES in your opinion.**	
4.8	***If 'Do you perform Pes measurements in critically ill patients?' is equal to 'yes' answer this question:***<br>I think that Pes could be helpful for setting PEEP in controlled ventilation	○ totally agree ○ agree ○ neither agree nor disagree ○ disagree ○ totally disagree
4.9	***If 'Do you perform Pes measurements in critically ill patients?' is equal to 'yes' answer this question:***<br>I think that Pes could be helpful for measuring lung and chest wall compliance	○ totally agree ○ agree ○ neither agree nor disagree ○ disagree ○ totally disagree
4.10	***If 'Do you perform Pes measurements in critically ill patients?' is equal to 'yes' answer this question:***<br>I think that Pes could be helpful for monitoring inspiratory lung stress in controlled ventilation	○ totally agree ○ agree ○ neither agree nor disagree ○ disagree ○ totally disagree
4.11	***If 'Do you perform Pes measurements in critically ill patients?' is equal to 'yes' answer this question:***<br>I think that Pes could be helpful for monitoring inspiratory lung stress in assisted ventilation	○ totally agree ○ agree ○ neither agree nor disagree ○ disagree ○ totally disagree
4.12	***If 'Do you perform Pes measurements in critically ill patients?' is equal to 'yes' answer this question:***<br>I think that Pes could be helpful for detecting patient-ventilatory asynchrony	○ totally agree ○ agree ○ neither agree nor disagree ○ disagree ○ totally disagree
4.13	***If 'Do you perform Pes measurements in critically ill patients?' is equal to 'yes' answer this question:***<br>I think that Pes could be helpful for measuring breathing effort	○ totally agree ○ agree ○ neither agree nor disagree ○ disagree ○ totally disagree
4.14	***If 'Do you perform Pes measurements in critically ill patients?' is equal to 'yes' answer this question:***<br>I think that Pes could be helpful for assessing the cause of weaning failure	○ totally agree ○ agree ○ neither agree nor disagree ○ disagree ○ totally disagree

4.15	***If 'Do you perform Pes measurements in critically ill patients?' is equal to 'yes' answer this question:***<br>I think that Pes could be helpful for measuring auto-PEEP during spontaneous breathing	◯ totally agree ◯ agree ◯ neither agree nor disagree ◯ disagree ◯ totally disagree
4.16	***If 'Do you perform Pes measurements in critically ill patients?' is equal to 'yes' answer this question:***<br>I think that Pes could be helpful for diagnosing inspiratoy muscle dysfunction	◯ totally agree ◯ agree ◯ neither agree nor disagree ◯ disagree ◯ totally disagree
4.17	***If 'Do you perform Pes measurements in critically ill patients?' is equal to 'yes' answer this question:***<br>I use Pes for<br>multiple options possible	☐ setting PEEP in controlled ventilation<br>☐ measuring lung and chest wall compliance<br>☐ monitoring inspiratory lung stress in controlled ventilation<br>☐ monitoring inspiratory lung stress in assisted ventilation<br>☐ detecting patient-ventilator asynchrony<br>☐ measuring breathing effort<br>☐ assessing the cause of weaning failure<br>☐ measuring auto-PEEP during spontaneous breathing<br>☐ diagnosing inspiratory muscle dysfunction<br>☐ research only<br>☐ other
4.17.1	***If 'I use Pes for' is equal to 'other' answer this question:***<br>Please specify other	

As you are not using esophageal pressure measurements, this form is not applicable. Please click 'Next'.

Use and implementation of advanced respiratory monitoring (Pes and EIT) in the ICU - Esophageal pressure - competence, experiences and team/organization

Number	Question	Answers

	**The following statements are about your competence and experience with Pes. For all statements, fill in what is applicable to Pes in your opinion.**	
5.1	***If 'Do you perform Pes measurements in critically ill patients?' is equal to 'yes' answer this question:***<br>It is clear to me how I should insert and fill the balloon	○ totally agree ○ agree ○ neither agree nor disagree ○ disagree ○ totally disagree
5.2	***If 'Do you perform Pes measurements in critically ill patients?' is equal to 'yes' answer this question:***<br>It is clear to me how I should verify the correct position of the balloon	○ totally agree ○ agree ○ neither agree nor disagree ○ disagree ○ totally disagree
5.3	***If 'Do you perform Pes measurements in critically ill patients?' is equal to 'yes' answer this question:***<br>It is clear to me how I should practically perform a Pes measurement in my patient	○ totally agree ○ agree ○ neither agree nor disagree ○ disagree ○ totally disagree
5.4	***If 'Do you perform Pes measurements in critically ill patients?' is equal to 'yes' answer this question:***<br>Pes measurements are too complex for me to perform	○ totally agree ○ agree ○ neither agree nor disagree ○ disagree ○ totally disagree
5.5	***If 'Do you perform Pes measurements in critically ill patients?' is equal to 'yes' answer this question:***<br>I know how to recognize artefacts in Pes tracings	○ totally agree ○ agree ○ neither agree nor disagree ○ disagree ○ totally disagree
5.6	***If 'Do you perform Pes measurements in critically ill patients?' is equal to 'yes' answer this question:***<br>I know how to ensure reliable tracings and measurements	○ totally agree ○ agree ○ neither agree nor disagree ○ disagree ○ totally disagree
5.7	***If 'Do you perform Pes measurements in critically ill patients?' is equal to 'yes' answer this question:***<br>I know how to calculate Pes-derived parameters	○ totally agree ○ agree ○ neither agree nor disagree ○ disagree ○ totally disagree
5.8	***If 'Do you perform Pes measurements in critically ill patients?' is equal to 'yes' answer this question:***<br>Pes measurements are too time-consuming for me to perform	○ totally agree ○ agree ○ neither agree nor disagree ○ disagree ○ totally disagree
5.9	***If 'Do you perform Pes measurements in critically ill patients?' is equal to 'yes' answer this question:***<br>Pes tracings are too complex for me to interpret	○ totally agree ○ agree ○ neither agree nor disagree ○ disagree ○ totally disagree

5.10	***If 'Do you perform Pes measurements in critically ill patients?' is equal to 'yes' answer this question:***<br>I can incorporate Pes measurements easily in my current workflow	○ totally agree ○ agree ○ neither agree nor disagree ○ disagree ○ totally disagree
5.11	***If 'Do you perform Pes measurements in critically ill patients?' is equal to 'yes' answer this question:***<br>I think Pes measurements are relevant for my patients	○ totally agree ○ agree ○ neither agree nor disagree ○ disagree ○ totally disagree
5.12	***If 'Do you perform Pes measurements in critically ill patients?' is equal to 'yes' answer this question:***<br>I think that Pes measurements need more validation before guiding treatment	○ totally agree ○ agree ○ neither agree nor disagree ○ disagree ○ totally disagree
5.13	***If 'Do you perform Pes measurements in critically ill patients?' is equal to 'yes' answer this question:***<br>What are you biggest considerations if you decide not to perform a Pes measurement?	☐ it takes too much time to perform<br>☐ we have too few equipment/materials available<br>☐ I have not enough experience in doing the measurements<br>☐ I am not sure how to interpret the values<br>☐ budget (too expensive to perform (disposables etc.))<br>☐ other<br>☐ not applicable
5.13.1	***If 'What are you biggest considerations if you decide not to perform a Pes measurement?' is equal to 'other' answer this question:***<br>Please specify 'other'	
	**The following statements are about your team and organization. For all statements, fill in what is applicable to Pes in your opinion.**	
5.14	***If 'Do you perform Pes measurements in critically ill patients?' is equal to 'yes' answer this question:***<br>My colleagues expect and support me to use Pes measurements	○ totally agree ○ agree ○ neither agree nor disagree ○ disagree ○ totally disagree
5.15	***If 'Do you perform Pes measurements in critically ill patients?' is equal to 'yes' answer this question:***<br>There are enough people at our department that can perform Pes measurements as intended	○ totally agree ○ agree ○ neither agree nor disagree ○ disagree ○ totally disagree

5.16	***If 'Do you perform Pes measurements in critically ill patients?' is equal to 'yes' answer this question:***<br>There are enough training materials for new Pes users in our department	◯ totally agree ◯ agree ◯ neither agree nor disagree ◯ disagree ◯ totally disagree
5.17	***If 'Do you perform Pes measurements in critically ill patients?' is equal to 'yes' answer this question:***<br>Our team evaluates the quality of Pes recordings of new users	◯ totally agree ◯ agree ◯ neither agree nor disagree ◯ disagree ◯ totally disagree
5.18	***If 'Do you perform Pes measurements in critically ill patients?' is equal to 'yes' answer this question:***<br>I can discuss my Pes findings with other experts when needed	◯ totally agree ◯ agree ◯ neither agree nor disagree ◯ disagree ◯ totally disagree
5.19	***If 'Do you perform Pes measurements in critically ill patients?' is equal to 'yes' answer this question:***<br>There is enough time to use Pes measurements in my day-to-day work	◯ totally agree ◯ agree ◯ neither agree nor disagree ◯ disagree ◯ totally disagree
5.20	***If 'Do you perform Pes measurements in critically ill patients?' is equal to 'yes' answer this question:***<br>Our department provides me with enough materials (disposables etc.) to use Pes measurements as intended	◯ yes<br>◯ no
5.21	***If 'Do you perform Pes measurements in critically ill patients?' is equal to 'yes' answer this question:***<br>Our department has local or standardized training materials available for learning how to perform Pes measurements (how to insert and calibrate the balloon, start the measurement)	◯ yes<br>◯ no
5.22	***If 'Do you perform Pes measurements in critically ill patients?' is equal to 'yes' answer this question:***<br>Our department has local or standardized protocols on when to perform Pes measurements (indications)	◯ yes<br>◯ no
5.23	***If 'Do you perform Pes measurements in critically ill patients?' is equal to 'yes' answer this question:***<br>Our department has local and standardized protocols on how to perform Pes measurements (how to do the measurement and interpret results)	◯ yes<br>◯ no
	**The following open questions are about clinical impementation of Pes.**	

5.24	***If 'Do you perform Pes measurements in critically ill patients?' is equal to 'yes' answer this question:*** In your experience, which factors will facilitate the use of Pes measurements in clinical practice?	
5.25	***If 'Do you perform Pes measurements in critically ill patients?' is equal to 'yes' answer this question:*** In your experience, what are the biggest concerns that hinder the use of Pes measurements in clinical practice?	
5.26	***If 'Do you perform Pes measurements in critically ill patients?' is equal to 'yes' answer this question:*** (Optional): What do you think should be the next steps towards a Pes-guided ventilation strategy?	
	**As you are not using Pes measurements, this form is not applicable. Please click 'Next'.**	

Use and implementation of advanced respiratory monitoring (Pes and EIT) in the ICU - Last remarks

Number	Question	Answers
6.1	Open question (optional): When do you prefer using Pes over EIT and vice versa?	

6.2	Room for any further comments or feedback	

Use and implementation of advanced respiratory monitoring (Pes and EIT) in the ICU - Outro

Thank you very much for contributing to the survey. Your participation will make a difference and will help us individualizing mechanical ventilation and improving patients' outcomes.

You can now close this survey.

Best regards,

Annemijn Jonkman, Mariangela Pellegrini, Gaetano Scaramuzzo, Jantine Wisse-Smit, Peter Somhorst and Erwin Ista
