# Supplementary material for: Clinical implementation of advanced respiratory monitoring with esophageal pressure and electrical impedance tomography: results from an international survey and focus group discussion
Source: Intensive Care Med Exp. 2024 Oct 21;12:93. doi: 10.1186/s40635-024-00686-9 (PMC11493933; doi:10.1186/s40635-024-00686-9)
Supplement: Supplementary file 2 — Supplementary material 2. [file 40635_2024_686_MOESM2_ESM.docx]

**Supplement 2**

******

***S2-Figure 1*** *Survey responses to the question: “I find that EIT or Pes helps me to….”. Answer options ranged from: totally agree, agree, neither agree nor disagree, disagree, totally disagree.*


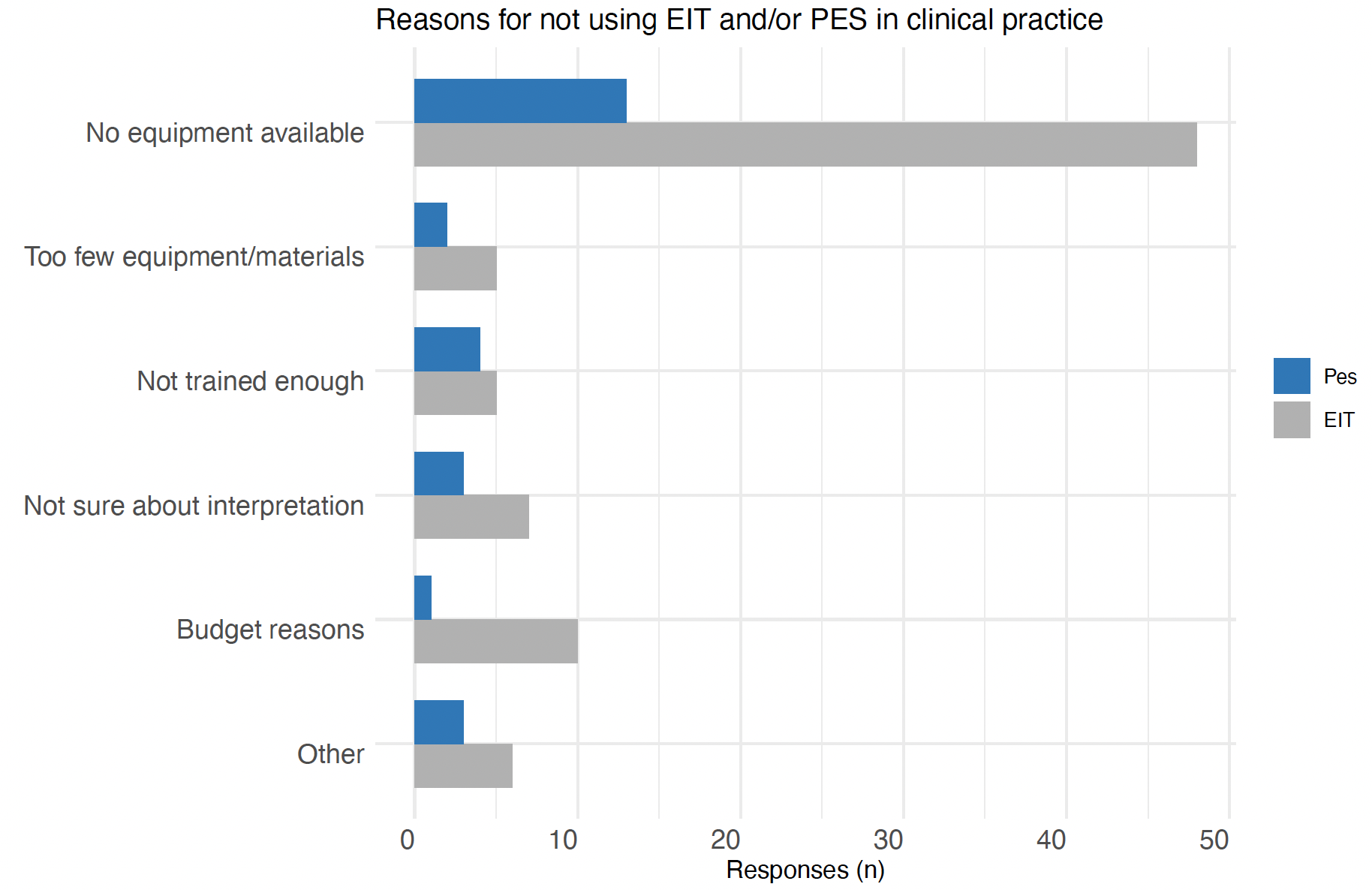


***S2-Figure 2*** *Survey responses to the questions: “Why do you not use Pes/EIT in your clinical practice?”. These questions were a follow-up question if respondents answered no to the questions: “Do you perform Pes/EIT measurements in critically ill patients?”. Multiple answers could be selected.*
